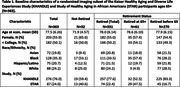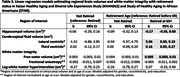# Association of retirement with MRI markers of vascular injury and neurodegeneration in a diverse cohort: Findings from KHANDLE and STAR

**DOI:** 10.1002/alz.094753

**Published:** 2025-01-09

**Authors:** Nancy X Chen, Yi Lor, Charles S. DeCarli, Lisa L. Barnes, Paola Gilsanz, Kristen M. George, Pauline Maillard, Elizabeth Rose Mayeda, M. Maria Glymour, Rachel A. Whitmer

**Affiliations:** ^1^ University of California, Davis, Davis, CA USA; ^2^ Department of Neurology & Imaging of Dementia and Aging Laboratory, University of California, Davis, Davis, CA USA; ^3^ Rush University Medical Center, Chicago, IL USA; ^4^ Kaiser Permanente Northern California Division of Research, Oakland, CA USA; ^5^ Department of Neurology and Center for Neuroscience, University of California, Davis, CA USA; ^6^ UCLA Fielding School of Public Health, University of California, Los Angeles, CA USA; ^7^ Boston University School of Public Health, Boston, MA USA

## Abstract

**Background:**

Prior research suggests that early retirement is associated with cognitive decline, but mechanisms are unknown. We investigated whether retirement is associated with MRI biomarkers of neurodegeneration and vascular injury in two diverse cohorts of adults ages 65+.

**Methods:**

The Kaiser Healthy Aging and Diverse Life Experiences and the Study of Healthy Aging in African Americans are harmonized longitudinal cohorts of racially/ethnically diverse adults who are long‐term Kaiser Permanente Northern California members. We included adults 65+ years and categorized baseline retirement status (retired vs not retired). Regional brain volumes and white matter (WM) integrity were measured using 3T MRI among a random subset of the harmonized cohorts. Hippocampal volume (cm^3^), lateral ventricle volume (cm^3^), third ventricle volume (cm^3^), and log‐WM hyperintensities (log‐WMH) (cm^3^) were normalized on intracranial volume and age at scan. We also examined free water fraction (FW) and fractional anisotropy (FA) normalized at age at scan. Linear regression models estimated associations of retirement status with regional brain volumes and WM integrity. All models adjusted for gender, race/ethnicity, and education. Sensitivity analyses examined retirement categorized as 1) retired before age 65, 2) retired at age 65+, and 3) not retired.

**Results:**

Participants’ (N = 363) mean age at scan was 77.5 (SD = 6.3), 56% were women, 50% had < college education, 24% identified as non‐Latino White, 91% were retired, of whom 82% retired before age 65 (Table 1). Retirement was not associated with MRI, whereas timing of retirement was. Adults who retired at 65+ had smaller hippocampal volume (b = ‐0.17, 95%CI ‐0.35,0.00), compared to those who retired before 65 (Table 2). Adults who retired at 65+ also had larger ventricular volumes (lateral ventricle: b = 5.06, 95%CI 0.93,9.19; third ventricle: b = 0.16, 95%CI 0.05,0.27) and higher FW (b = 0.005, 95%CI 0.000,0.01) than those who retired before 65 (Table 2).

**Conclusion:**

Retiring at 65+ appeared detrimental to late‐life brain health in this diverse cohort of older adults, contrary to the cognitive decline literature. Future studies should explore factors contributing to the impact of later retirement on late‐life brain health.